# Distribution and antifungal susceptibility profiles of *Candida* species isolated from candidemia patients admitted to Egyptian tertiary hospitals: a cross-sectional study

**DOI:** 10.1186/s12879-024-10007-w

**Published:** 2024-10-18

**Authors:** Heba Sherif Abdel Aziz, Dalia Kadry Ismail, Nessma Sayed Ahmed Mohammed, Marwa O. Elgendy, Dina M. Bassiouny

**Affiliations:** 1 Clinical and Chemical Pathology Department, Faculty of Medicine, Cairo University, Cairo, Egypt; 2 Clinical Pharmacy Department, Faculty of Medicine, Beni-Suef University Hospitals, Beni-Suef University, Beni-Suef, Egypt; 3 Clinical Pharmacy Department, Faculty of Pharmacy, Nahda University, Beni-Suef, Egypt

**Keywords:** Candidemia, MALDI-TOF MS, VITEK, Antifungals, Resistance

## Abstract

**Background:**

Candidemia is a widespread threat that can lead to significant complications in healthcare settings.

**Objectives:**

Our study aimed to identify isolates of *Candida* isolated from blood culture bottles of patients with candidemia and assess their antifungal susceptibility profiles.

**Methods:**

We conducted a cross-sectional study at Cairo University tertiary care hospitals over 16 months including 90 patients. *Candida* isolates were collected from blood culture bottles, and identified using MALDI-TOF MS technology of VITEK MS PRIME (bioMérieux) with the corresponding database VITEK IVD Database 3.2. followed by antifungal susceptibility testing using VITEK 2 Compact system.

**Results:**

*Candida albicans* was the most common species isolated from both pediatric and adult patients with percentages of 47.3% and 36.4% respectively, followed by *Candida parapsilosis* with percentages of 32.6% and 25.0% respectively. Voriconazole showed the highest antifungal activity at 90.9% of isolates in adults and 95.7% in pediatrics, followed by caspofungin and micafungin. The mean hospital stays for adults ranged from 8 to 30 days and from 10 to 42 days in the pediatric group.

**Conclusions:**

*C. albicans* remains the predominant species isolated from both pediatric and adult candidemia patients, despite a notable increase in other species. *C. tropicalis* and *C. parapsilosis* are considered the most common non-*albicans Candida* (NAC) species. The rise in *Candida* species other than *albicans* highlights the urgent need for effective antifungal stewardship programs. Voriconazole exhibited the higher antifungal activity followed by caspofungin and micafungin.

## Background

*Candida* species constitute a major part the microflora of the human skin, mucous membranes, and digestive tract [1]. However, *Candida* can cause mild or severe infections in humans and is being a notable cause of bloodstream infection (BSI) [2, 3]. The risk factors for *Candida* BSI include ICU admission with prolonged hospital stay, central venous line insertion, antibiotic over use, and immunosuppression [4]. Although *C. albicans* is the most common species causing BSI, other species of *Candida* are getting more attention in patients with hematological diseases, immunosuppressed, or ICU admitted [5]. Intrinsic and acquired resistances to antifungal agents are often reported in non*-albicans Candida* (NAC) species [5, 6]. The distribution of candida species and antifungal resistance rate vary geographically; therefore, updated data of the species distribution and antifungal susceptibility pattern of NAC is needed to enhance the treatment plan of candidemia and detect the mechanism of drug resistance [7]. 

There are different laboratory methods for identification of the various pathogenic *Candida* spp. Conventional methods, which include biochemical tests and morphological studies, are less practical nowadays. Automated systems such as VITEK 2 Compact can provide rapid and accurate identification and antifungal susceptibility results as well [8]. 

Matrix-assisted laser desorption/ionization-time of flight mass spectrometry (MALDI-TOF MS) is another automated method that can be used for yeast and other organisms’ identification from culture plates. It has been approved as an accurate identification method of various yeast species [9, 10]. 

This study aimed at identification of *Candida* isolates, collected from blood culture bottles of patients with candidemia, using MALDI-TOF MS and testing for antifungal susceptibility using VITEK 2 Compact followed by analysis of the results to get a clearer image of distribution of different species of *Candida* in Egypt and the current antifungal susceptibility pattern among the studied group to be used in modification in future management plans.

## Methods

### Ethical approval

This study acquired an approval letter from the research ethical Committee of the Faculty of Medicine, Cairo University (Serial: MS-614-2021).

### Study design and settings

This cross-sectional study was conducted at Tertiary care hospitals of Cairo University. Adult and pediatric patients admitted from October 2021 to January 2023, whose blood cultures revealed *Candida* species, were enrolled in the study.

### Sample size

The sample size was calculated to determine the minimum appropriate sample size for the prevalence of different *candida* species in patients with candidemia. According to the literature, the prevalence of *C. albicans* in similar situations ranged from 20.1 to 32%, and non-*albicans* prevalence ranged from 68 to 79.5% [11, 12] with an average of *C. albicans* of 26% ± 6%. If we assumed that this was the true population prevalence, a minimum of 88 participants were enrolled in the study to be able to reject the null hypothesis with 80% power, setting the alpha error to 0.05 using the generic Z test.

### Methodology and data collection

Ninety patients were enrolled and data from these patients were collected and included age, sex, department of admission, clinical diagnosis, operation if performed, risk factors (e.g. chemotherapy, radiotherapy, and diabetes mellitus), type of inserted devices, length of hospital stay, clinical outcome of the patient.

Microbiological work started with the withdrawal of blood culture samples from the affected patients, followed by processing till performing antifungal susceptibility testing.

### Blood culture samples collection

Under complete aseptic technique, blood culture samples were collected and inoculated in the Bactec or Bact/Alert blood culture bottles and labelled with the patient data.

### Processing of positive blood culture bottles

Blood culture bottles with positive alarm for microbial growth, were submitted for direct Gram stain and were sub cultured on blood, chocolate, and MacConkey agar plates. Agar plates of blood, chocolate and MacConkey were incubated at 37° C for 24 h. White creamy growth on blood agar and chocolate agar that showed Gram positive oval yeast cells with single or multiple budding were provisionally considered *Candida* spp.

### Identification of the species of *Candida*

Identification of the type of *Candida* was done by MALDI-TOF MS technology of VITEK MS PRIME (bioMérieux) with the corresponding database VITEK IVD Database 3.2.which uses software that compares the spectral profile of the unknown organism with a reference database [13]. 

It has to be noted that *Candida krusei* will be used for *Pichia kudriavzevii* and *Candida glabrata* for *Nakaseomyces glabrata* [1]. 

### Anti-fungal susceptibility testing

Automated testing of anti-fungal susceptibility of the *Candida* isolates was performed by VITEK 2 Compact using antifungal cards *(*bioMérieux, France) to measure the minimal inhibitory concentration (MIC) of the included panel of antifungals. The tested antifungal panel included amphotericin B, voriconazole, caspofungin, fluconazole, flucytosine, and micafungin [14]. 

### Statistics analysis

The statistical package for the Social Sciences (SPSS) version 28 (IBM Corp., Armonk, NY, USA), was used for data coding. Data summarization was done using mean, median, standard deviation, maximum and minimum in quantitative data and using. For categorical data, frequency (count) and relative frequency (percentage) were used. Quantitative variables were subjected to comparison using the non-parametric Kruskal-Wallis and Mann-Whitney tests [15]. Chi square (χ2) test was performed for comparing categorical data. When the expected frequency is less than 5, exact test was used instead [16]. Agreement between categorical variables was tested using Kappa measure of agreement. P-values less than 0.05 were considered statistically significant.

## Results

Adult and pediatric patients had an age mean of 47.45 years and 24.46 months respectively. Female patients were more than males in the adult group with percentages of 59.1% and 40.9% respectively. The pediatric group showed equal percentages of females and males.

Regarding the comorbidities, among the adult patients, 29.5% had diabetes mellitus (DM) and hypertension (HTN). None of the pediatric patients had comorbidities such as DM or HTN.

Immunosuppression was present in 6.8% of the adult patients and 10.9% of the included children.

The clinical characteristics of the enrolled patients, regarding the department of admission and the cause of the admission according to the affected system, were summarized in Table 1.

**Table 1:** Clinical characteristics of the enrolled patients

		Adults (*n* = 44)		Pediatrics (*n* = 46)	
		Count	%	Count	%
Department	Medical ICU	11	25.0%	17	37.0%
	Surgical ICU	27	61.4%	13	28.3%
	Ward	6	13.6%	16	34.8%
Cause of admission according to the affected System	General	0	0.0%	2	4.3%
	Gastrointestinal	14	31.8%	9	19.6%
	Respiratory	2	4.5%	7	15.2%
	Neurology	10	22.7%	9	19.6%
	Cardiovascular	11	25.0%	12	26.1%
	Orthopedic	2	4.5%	0	0.0%
	Renal	1	2.3%	4	8.7%
	Plastic surgery	3	6.8%	1	2.2%
	Hematological	0	0.0%	2	4.3%
	Ophthalmology	1	2.3%	0	0.0%

In the adults’ group, 13 patients (29.5%) had undergone surgery. Among those who had undergone surgery, the most affected system in the adults was the gastrointestinal tract (11.4%), followed by the orthopedic (6.8%) and the plastic surgery (6.8%).

In the pediatric group, 8 patients (17.4%) had undergone surgery. The most affected system was the cardiovascular (13.0%), followed by plastic surgery (2.2%) and neurology (2.2%).

Medical devices were used in 22.7% of adult patients and in 34.8% of pediatric patients. The distribution of the used medical devices showed that mechanical ventilation was used in 11.4% of adult patients and in 26.1% of pediatric patients. The central venous line was inserted in 4.5% of adult patients and no central venous line was inserted in the pediatric patients.

The most prevalent species identified was *C. albicans* (36.4%), followed by *C. tropicalis* (25.0%) and *C. parapsilosis* (25.0%). In the pediatric patients, *C. albicans* was also the most prevalent species identified (47.8%), followed by *C. parapsilosis* (32.6%). Table 2.

**Table 2:** Distribution of different *Candida* species in the studied population

		Adults (*n* = 44)		Pediatrics(*n* = 46)	
		Count	%	Count	%
*Candida* Species identified by MALDI-TOF	***C. albicans***	16	36.4%	22	47.8%
	C. auris	3	6.8%	0	0.0%
	C. glabrata	1	2.3%	1	2.2%
	C. guilliermondii	1	2.3%	0	0.0%
	C. krusei	0	0.0%	2	4.3%
	C. lusitaniae	0	0.0%	1	2.2%
	C. metapsilosis	1	2.3%	0	0.0%
	C. parapsilosis	11	25.0%	15	32.6%
	C. tropicalis	11	25.0%	5	10.9%

The highest antifungal susceptibility was reported to voriconazole with a percentage of 90.9% in the adults and 95.7% in the pediatric followed by caspofungin which was 88.6% in the adults and 91.3% in the pediatrics Table 3.

**Table 3:** The results of antifungal susceptibility of tested *Candida* isolates

		Adults (*n* = 44)		Pediatrics (*n* = 46)	
		Count	%	Count	%
Fluconazole	Susceptible	36	81.8%	36	78.3%
	Resistant	6	13.6%	8	17.3%
	SDD	2	4.5%	2	4.3%
Voriconazole	Susceptible	40	90.9%	44	95.7%
	Resistant	4	9.1%	2	4.3%
Caspofungin	Susceptible	39	88.6%	42	91.3%
	Resistant	5	11.4%	1	2.2%
	Intermediate	0	0.0%	3	6.5%
Micafungin	Susceptible	40	90.9%	40	87.0%
	Resistant	4	9.1%	2	4.3%
	Intermediate	0	0.0%	4	8.7%
Amphotericin B	Susceptible	39	88.64%	33	71.7%
	Resistant	3	6.82%	8	17.4%
	Intermediate	2	4.54%	5	10.9%
Flucytosine	Susceptible	39	88.6%	42	91.3%
	Resistant	5	11.4%	4	8.7%

*C. albicans* and *C. tropicalis* were Susceptible the most to voriconazole and fluconazole. *C. parapsilosis* showed the highest susceptibility to caspofungin. Table 4.

**Table 4:** The antifungal susceptibility testing results of different *Candida* species in the whole studied population

Type of Candida Spp.	Fluconazole		Voriconazole		Caspofungin		Micafungin		Amphotericin B		Flucytosine	
	Count	%	Count	%	Count	%	Count	%	Count	%	Count	%
*C. albicans* (*n* = 39)	37	94.8	37	94.8	34	87.1	33	89.1	32	82	37	94.8
*C. tropicali* (*n* = 15)	15	100	15	100	13	86.6	14	93.3	13	86.6	13	86.6
*C. parapsilosis* (*n* = 26)	16	61.5	23	88.4	26	100%	25	96.1	19	73	22	84.6
*C. auris* (*n* = 3)	0.0	0.0	0	0.0	0.0	0.0	0	0.0	0.0	0.0	0.0	0.0
*C. guilliermondii* (*n* = 1)	1	100	1	100	1	100	1	100	1	100	1	100
*C. glabrata* (*n* = 2)	0.0	0.0	2	100	1	50	1	50	1	50	2	50
*C. metaapsilosis* (*n* = 1)	1	100	1	100	1	100	1	100	1	100	1	100
*C. krusei* (*n* = 2)	0	0.0	1	50	1	50	1	50	1	50	0	0.0
*C. lusitaniae* (*n* = 1)	1	100	1	100	1	100	1	100	1	100%	1	100

*C. auris* has been found to be resistant to all antifungals. This makes the infection with this species a serious concern and increases the need for proper infection control measures in healthcare settings.

Moreover, *C.parapsilosis* showed a high resistance rate to azoles specially fluconazole. This gives more attention to this species that is being resistant to one of the safest and the most popular antifungals.

The mean length of hospital stay ± SD for the adult patients was 20.34 ± 12.74 days, while the mean length of hospital stay ± SD for the pediatric patients was 20.37 ± 10.50 days.

Regarding the outcome of the included patients, 54.5% of the adult patients were discharged and 45.5% died, while in the pediatric group, 58.7% were discharged and 41.3% died.

Among the adult patients, the mean the length of hospital stay varied from 8 days for *C. metapsilosis* to 30 days for *C. guilliermondii*. However, there was no significant relation between the length of hospital stay and the different isolated *Candida* species (p value: 0.584).

Among the paediatric patients, the mean of the length of hospital stay varied from 10 to 42 days, with the highest mean length of stay observed in cases of *C. krusei*. However, no significant relation was found. (P value: 0.237)

## Discussion

Candidemia is a critical hematogenous infection caused by the *Candida* species that predominantly impacts individuals with compromised immune systems, such as those treated with chemotherapy or those diagnosed with HIV/AIDS. Early identification is crucial for proper management and better patient outcomes [17]. 

The global and temporal distribution of *Candida* species responsible for candidemia exhibits variability, with *C. albicans* historically being the predominant species isolated. The frequency of non-*albicans Candida* species, namely *C. glabrata*,* C. tropicalis*, and *C. parapsilosis*, has escalated in recent times, leading to the emergence of antifungal-resistant strains [18].

Our aim in our current study was to assess the frequency and antifungal susceptibility of various *Candida* species responsible for candidemia in adult and pediatric patients who were admitted to tertiary hospitals affiliated with Cairo University.

In the present study, the median age of adult patients was 47 years and 2 years for pediatric patients and that was near to a similar study conducted in USA that showed a median adult age of 65 years old and 1 year for pediatric patients [19]. 

The management of candidemia in the ICU is complexed. In fact, candidemia is associated with 47% mortality, with higher rates in critically ill patients [20]. 

In the present study, the majority of adult patients were admitted to the surgical ICU (61.4%), while the majority of pediatric patients were admitted to the medical ICU (37.0%). That was near to a similar study conducted a tertiary care hospital in Western India in which most of included patients were ICU patients in a percentage of 78% of the total cases [21]. 

Comorbidities including chronic illnesses, bacterial infections, assisted ventilation, catheterization, and ICU admission have increased the risk of getting infected by different *Candida* species in different body sites [22]. 

In the current study, among the adult patients, the most prevalent comorbidities were diabetes mellitus and hypertension, with a prevalence of 29.5% for both and that was near to a study conducted in private sector hospital in Western India, that showed diabetes was reported in 46% of patients, and hypertension in 37% of the studied patients [21]. 

Another study, conducted in United States, showed similar comorbid conditions among included patients with systemic candidiasis with a percentage of 39% of hypertensive patients, and 26% diabetic patients [23]. 

In our study, ventilation as one of the risk factors for systemic candidiasis contributed to 10% of included adult patients and that was similar to a prospective observational study, carried out in the respiratory ICU of a tertiary care level respiratory institute from 2015 to 2016 that showed 10% invasive candidiasis in ventilated patients [24]. 

*Candida* species distribution changes continuously according to geographic locations, patient population and the used antifungal drugs. It was reported that the use of fluconazole increases breakthrough infection with *C. krusei* and *C. glabrata*. On the other hand, administration of caspofungin, to a lesser extent, increases fungal infection by *C. glabrata*, *Candida parapsilosis*, and *Candida krusei* [25]. Therefore, up-to-date epidemiological data of *Candida* species distribution and antifungal susceptibility profiles to commonly used antifungal agents are crucial to report to serve as a guide for clinicians in management plans [26]. 

In our present study, *C. albicans* was the most common type isolated with a percentage of 36% followed by *C. tropicalis* (25.0%) and *C. parapsilosis* (25.0%) in adult patients. In the pediatric group, *C. albicans* was also the most common identified (47.8%), followed by *C. parapsilosis* (32.6%). These percentages were near to a similar study conducted in China, Anhui Medical University hospital, in which *C. albicans* was isolated from 42.17% of enrolled patients [27]. Moreover, as shown by many research papers, *C. albicans* remains the main cause and the most commonly isolated from samples of patients in different geographical regions and countries including the United States having the biggest percentage of 67%, followed by Italy 61/2% then Saudi Arabia (38.3%), China (36.1%), Egypt (36%) and Kuwait (32%) [28–33]. 

The most common type of non-*albicans Candida* isolated from our patients was *C. tropicalis* with a percentage of 25% of adult patients and that was similar to the study conducted in China that showed *C. tropicalis* contributing to 21% of the total isolated non-*albicans Candida* [27]. , while disagreement was reported in another study that was conducted in Israel and showed *C. glabrata* as the most prevalent *NAC* type in adult patients with candidemia (40%) [34]. 

Regarding antifungal susceptibility results, sensitivity to caspofungin as same as micafungin was 93.2% in the adults, while sensitivity to caspofungin was 91.3% in the pediatrics. That was near to a similar study conducted in China that showed *C. albicans* and non-*albicans Candida* with 100% sensitivity to caspofungin [27]. 

Resistance to voriconazole was noted in 2% of included adult patients and that was near to the study conducted in China that showed 1% voriconazole resistance of *C. albicans* and 1% in *C.* non *albicans* [27]. 

Being an easy commercial method, the Vitek 2 system is being used broadly for antifungal susceptibility testing of Candida spp.

On the other hand, data about Vitek2 compact performance against C. auris are limited and revealed disagreements in some papers. A comparison conducted between commercial antifungal testing methods like Vitek2 Compact and reference methods of CLSI and broth microdilution using 100 isolates of C.auris, showed poor CLSI-Vitek 2 agreement for Amphotericin B (29%) [35].

The mean hospital stay duration for adult and pediatric patients in our study was 20 days, similar to a study conducted in USA that showed an average hospital stay of 18.6 days to included adult patients and 21 days for pediatric patients with candidemia [19]. 

## Conclusions

*Candida albicans* remains the predominant species isolated from both pediatric and adult candidemia patients, despite a notable increase in the species other than *albicans*. *C. parapsilosis and C. tropicalis* are the most prevalent non-*albicans Candida* species. The rise in uncommon *Candida* species highlights the urgent need for effective antifungal stewardship programs. The highest antifungal susceptibility was found to voriconazole, followed by caspofungin and micafungin.

### Recommendations

Application of similar studies on a wider scale with a larger sample size will be beneficial to get better data about the current *Candida* distribution and antifungal susceptibility profiles.

## Data Availability

The datasets used and/or analyzed during the current study are available from the corresponding author on reasonable request.
